# Massive Bilateral Pulmonary Embolism in a Healthy 37-Year-Old Male: A Case of Atypical Presentation

**DOI:** 10.7759/cureus.78107

**Published:** 2025-01-27

**Authors:** Salah A Mustafa, Ehab M Abbas, Essa A Alkhalifa, Ali S Buallay

**Affiliations:** 1 General Practice, Arabian Gulf University, Manama, BHR; 2 Emergency Medicine, Bahrain Defence Force Hospital - Royal Medical Services, Riffa, BHR; 3 Internal Medicine, Bahrain Defence Force Hospital - Royal Medical Services, Riffa, BHR

**Keywords:** atypical presentation, differential diagnosis, early diagnosis, hemodynamic instability, massive pulmonary embolism, pulmonary embolism, risk factors

## Abstract

Pulmonary embolism (PE) is a potentially fatal condition caused by the obstruction of pulmonary arteries by a blood clot, commonly originating from deep vein thrombosis (DVT). Although traditional risk factors include recent surgery, immobility, cancer, or thrombophilia, PE can also occur in patients without these predisposing factors, presenting significant diagnostic challenges. In this case report, we present a 37-year-old Pakistani male with no significant medical history who suffered a massive PE. The patient experienced an initial collapse at home and a second collapse en route to the hospital, necessitating cardiopulmonary resuscitation (CPR). Despite the absence of conventional risk factors, emergent diagnosis and prompt treatment, including thrombolysis and anticoagulation, led to a positive outcome. This case underscores the importance of maintaining a high index of suspicion for PE, even in atypical presentations. It emphasizes the need for clinicians to include PE in the differential diagnosis for sudden collapse, ensuring timely intervention and improved survival rates.

## Introduction

Acute pulmonary embolism (PE) is a common and life-threatening condition that results from a blood clot that develops within the blood vessel and travels to the arteries that supply the lung, ending with a blockage of the artery and could lead to death. Timely recognition and treatment are crucial due to its high morbidity and mortality rates. Early intervention is essential for improving patient outcomes [1]. Symptoms of PE range from asymptomatic cases to chest pain, shortness of breath, hemoptysis, and episodes of fainting or collapse, making timely diagnosis challenging [2]. Bedside lung ultrasound is a useful diagnostic tool for managing patients with suspected PE. While it is not the gold standard for diagnosis, it can be especially helpful when CT pulmonary angiography (CTPA) is contraindicated or unavailable, with a reported sensitivity of 87.0% and specificity of 81.8% [3-4]. Furthermore, the Wells score, PE rule-out criteria, D-dimer testing, and imaging techniques like CT pulmonary angiography are crucial for accurately assessing and confirming the diagnosis of PE [5]. Anticoagulation is the primary treatment for managing and preventing venous thromboembolism, including deep vein thrombosis and PE [6]. Diagnosing PE is challenging, especially when symptoms are vague or non-specific. Misdiagnosis can lead to severe morbidity or even death [7]. Computed tomographic pulmonary angiography (CTPA) with multidetector-row technology is the preferred imaging method for suspected PE [8]. The Bova Score is a vital tool for identifying high-risk PE patients and predicting short-term outcomes. It incorporates clinical parameters such as vital signs, right ventricular dysfunction, troponin levels, and, as highlighted in a recent study, plasma galectin-3 (Gal-3). Gal-3 has been shown to rapidly and accurately diagnose acute PE, helping guide treatment decisions and determine the appropriate intensity of care [9-10]. This case report highlights the complex presentation and management of a massive bilateral PE in a patient without typical risk factors, highlighting the importance of the diagnosis and personalized treatment for positive outcomes.

## Case presentation

A 37-year-old Pakistani male, with no significant medical or surgical history, received two doses of the Sinopharm COVID-19 vaccine in March and April 2021, tested positive for COVID-19 in May 2021, and got a Pfizer booster in December 2021. He was hospitalized for eight days after collapsing at home with symptoms of dizziness, shortness of breath, and sweating. Despite no prior health issues or allergies, he collapsed again upon paramedics' arrival, requiring CPR. In the emergency department, he presented with low oxygen saturation (85%) and hypotension (80/40 mmHg) (Table 1), which improved after intravenous fluids. The absence of typical PE risk factors and recent medical events made diagnosis difficult. After stabilization with the ABCDE approach, oxygen, and fluids, his vital signs upon admission are presented in Table 1.

**Table 1:** The patient vitals on arrival

Patient’s vitals	
Blood Pressure	85/40 mmHg
Heart Rate (beats per minute)	100
SpO_2_ (on room air)	91%
Respiratory Rate (breaths per minute)	28

The physical examination revealed that the patient was drowsy and sweating profusely. His pupils were round and reactive to light, with no signs of tongue biting, jaundice, or neck rigidity. There was no pedal edema. Chest examination showed reduced air entry and crepitations, but heart sounds (S1 and S2) were normal. The abdomen was lax and non-tender, and bowel sounds were present. No pain, tenderness, swelling, warmth, redness, or discoloration in the calves and no focal neurological deficits were noted. A series of diagnostic tests were performed (Table 2).

**Table 2:** The patient labs on arrival

Test	Results	Reference Range
Red blood cells (x10^12^/L)	5.2	4.00-5.50
White blood cells (x10^9^/L)	16.69	4.00-11.00
Neutrophils (x10^9^/L)	14.01	1.50-8.00
Monocytes (x10^9^/L)	1.02	0.20-1.00
Hemoglobin (g/L)	14.9	13.0-18.0
HCT (L/L)	0.435	0.40-0.50
MCV (fL)	83.7	80.00-100.00
Platelets (x10^9^/L)	12.7	150.00-450.00
PT (sec)	14.7	13.00-15.00
INR (sec)	1.07	0.98-1.13
Appt (sec)	33	28.00-45.00
D-dimmer (ug/ml)	2.48	0.00-0.50
Troponin (ng/mL)	0.057	0.00-25.00
Blood Sugar (mmol/L)	7	4.11-5.89
VBG		
Ph	7.387	7.350-7.450
pCO_2_ (mmHg)	35.7	32.0-48.0
pO_2_ (mmHg)	47.5	83-108
HCO_3_ (mmol/L)	21.0	22-28

**Figure 1:**
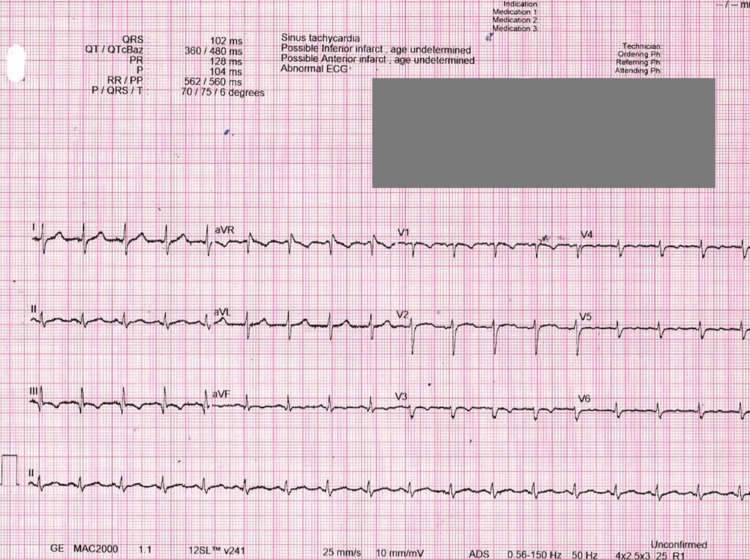
Initial ECG on arrival to the hospital

The echocardiogram (Figure 1) revealed sinus tachycardia with an S1 Q3 T3 pattern and a heart rate of 107 bpm, along with right heart dilation, massive PE, and right ventricular strain, all suggestive of PE. Random blood sugar (RBS) was measured at seven mmol/L. Venous blood gas (VBG) analysis indicated a pH of 7.38, pCO_2_ at 35 mmHg, HCO_3_ at 21 mmol/L, oxygen saturation of 83%, potassium level of 4 mmol/L, and hemoglobin at 15 g/dL. The patient's troponin level was elevated at 0.057 ng/mL. Coagulation profile results showed positive D-dimer levels of 2.48 µg/mL, with INR at 1.07, a PT of 14.7 seconds, and an APTT of 33 seconds (Table 2).

**Figure 2:**
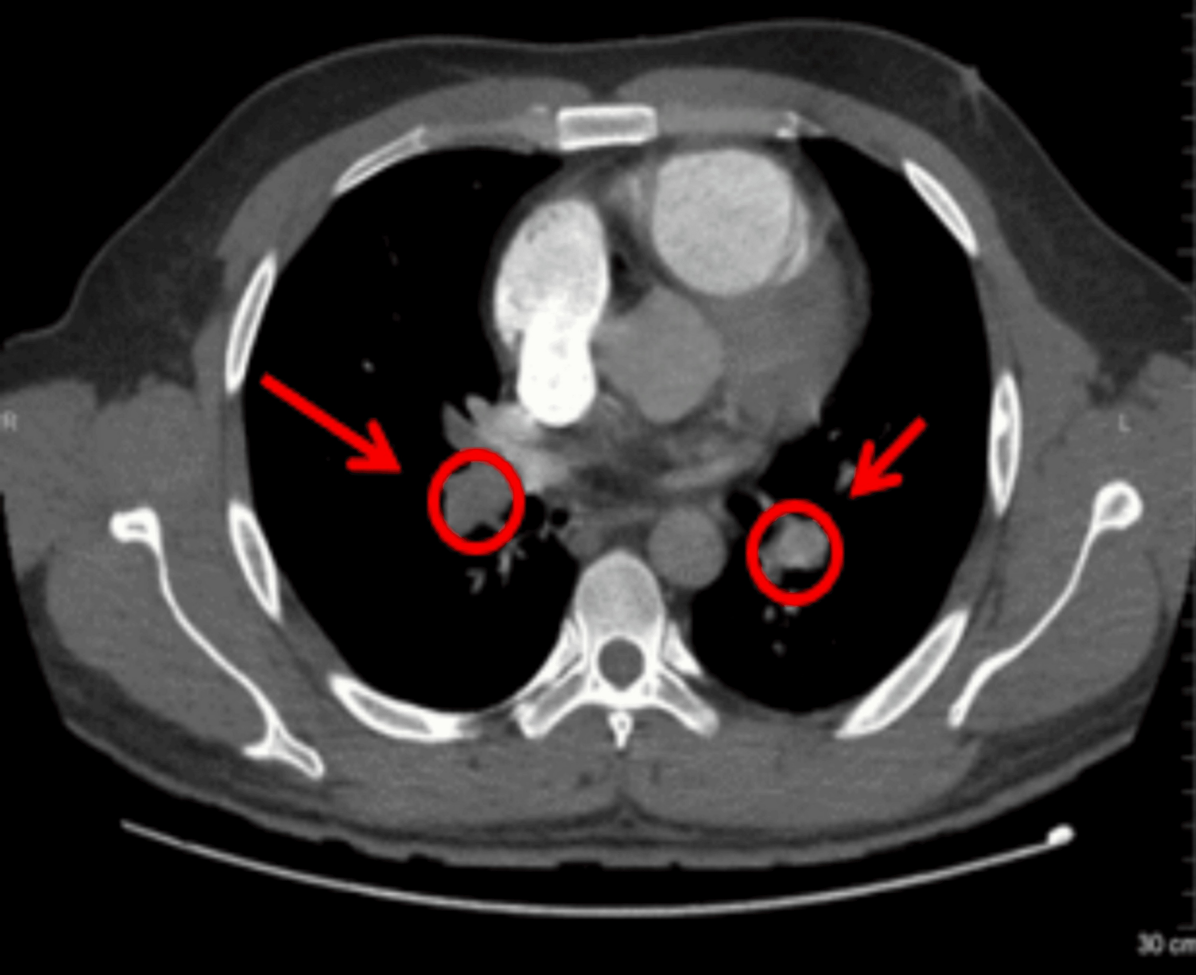
The CT pulmonary angiogram demonstrates a filling defect within the right and left pulmonary arteries, consistent with a massive bilateral pulmonary embolism The thrombus is visible as an area of low attenuation in the main pulmonary arteries, showing obstruction of blood flow.

**Figure 3:**
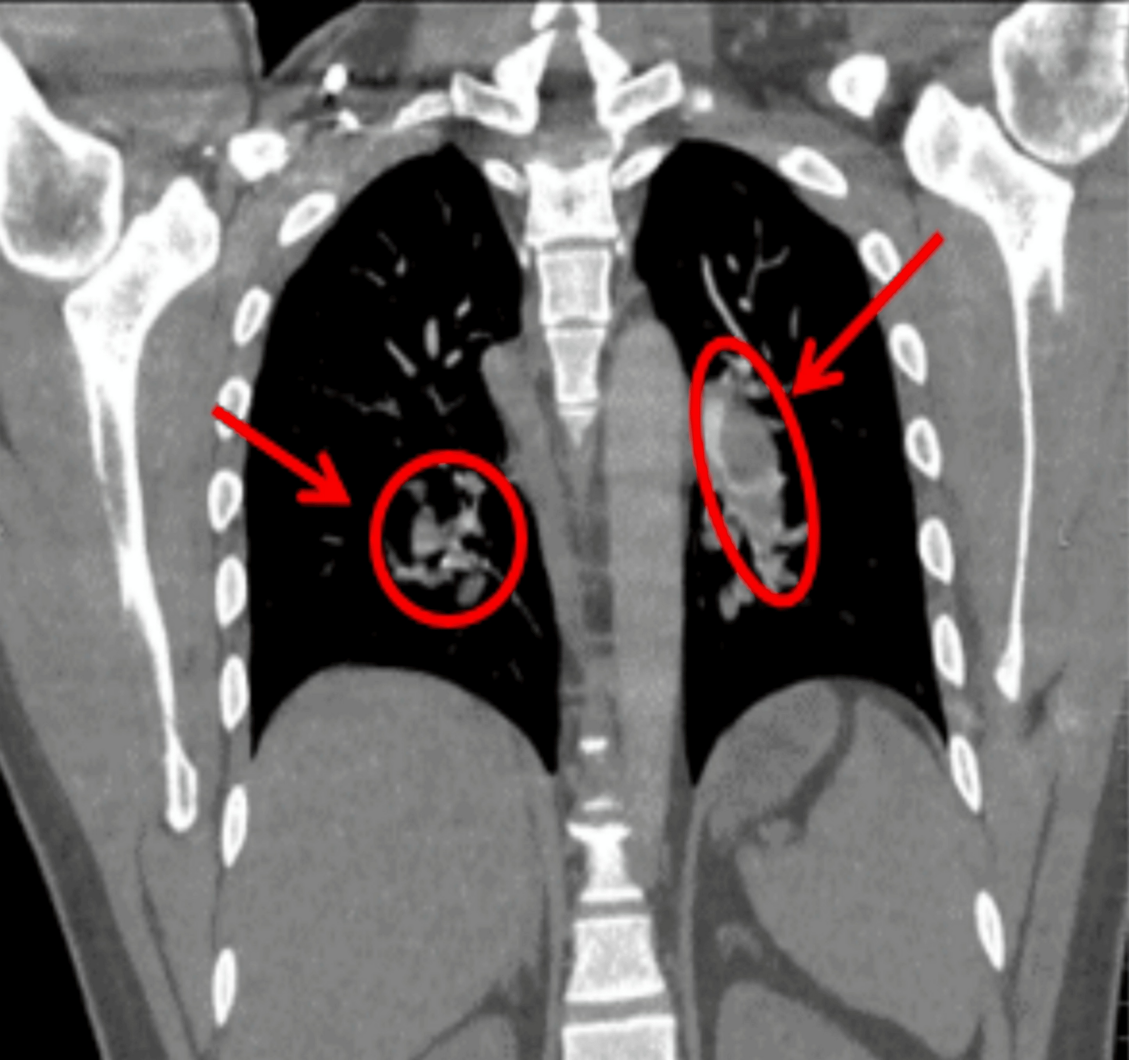
The CT pulmonary angiogram demonstrates filling defects within the right and left pulmonary arteries, extending into their branches and consistent with bilateral pulmonary emboli

**Figure 4:**
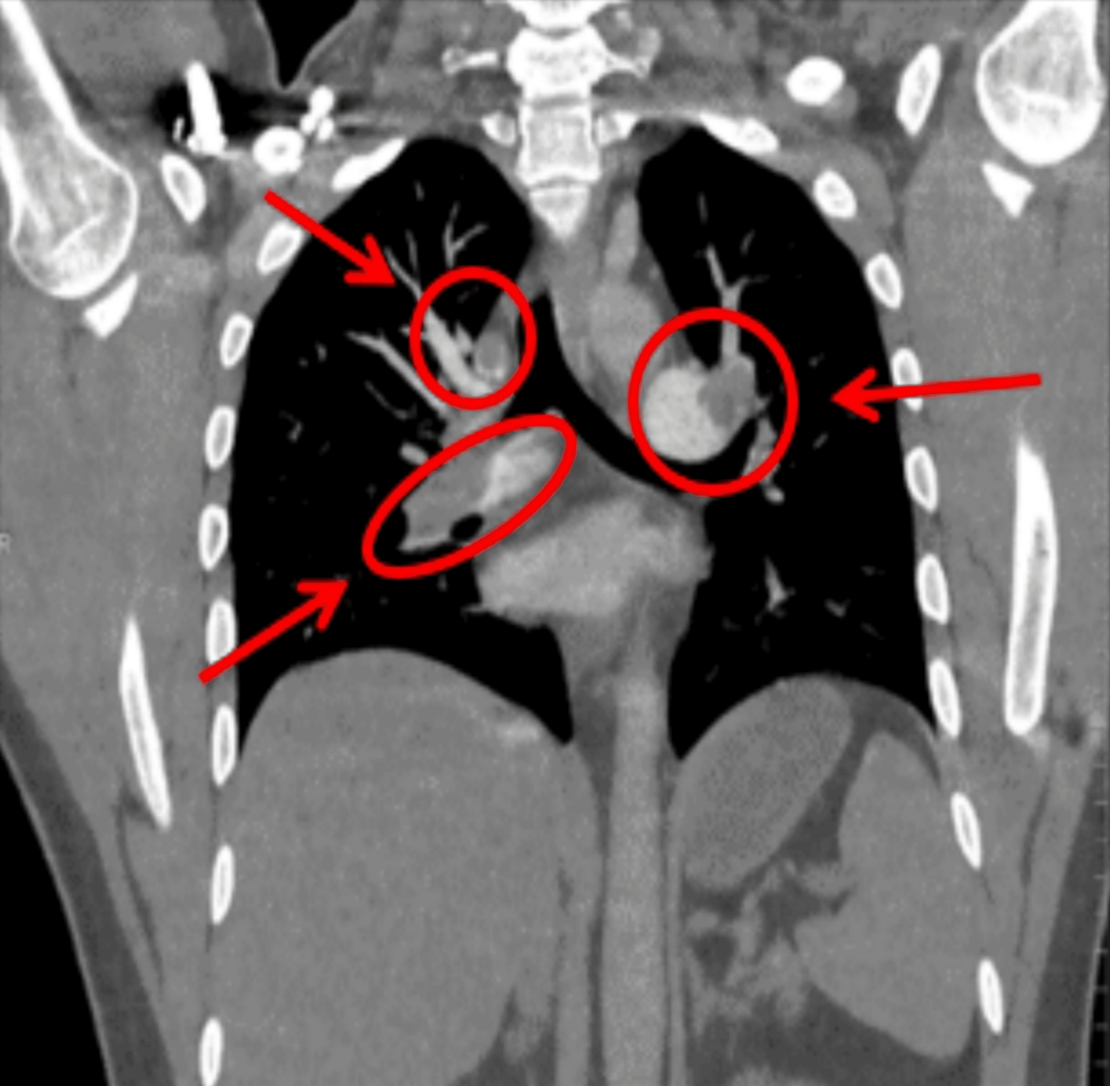
The CT pulmonary angiogram shows prominent filling defects in both the right and left pulmonary arteries, confirming the presence of bilateral pulmonary emboli The thrombi are within the central pulmonary arteries and extend into their proximal branches.

Based on the CT pulmonary angiogram (CTPA), filling defects were observed in the right and left main pulmonary arteries, indicating the presence of thrombi (Figure 2). The thrombus extended into the right intra-lobar artery, including the middle and lower branches (Figure 3), and the left interlobular artery and its branches (Figure 4). The CTPA revealed a massive bilateral PE, with filling defects in the bilateral main pulmonary arteries and segmental and sub-segmental branches, consistent with a large PE. Additionally, a 2 cm nodule in the right lower lobe of the lung seen in the X-ray was identified as a hamartoma related to the PE, with no abnormalities in the brain. The patient was kept on a central line in the emergency room, and fluid hydration started, which was carefully monitored. The patient was started on a thrombolytic tissue plasminogen activator (tPA) protocol with no contraindications and was stabilized before being moved to the intensive care unit (ICU) at midnight.

On the second day, the patient remained in the ICU on a strict heparin infusion, closely monitoring, activated partial thromboplastin time (APTT), vital signs, and signs of bleeding. His vital signs included a blood pressure of 99/68 mmHg, heart rate of 106 bpm, temperature of 36.8°C, respiratory rate of 13, and oxygen saturation of 97% on 2 L of nasal cannula (NC). As part of the workup to rule out other causes or risk factors that could have contributed to the development of PE in our patient, a decision was made to proceed with a lower limb Doppler study and a thrombophilia screen to assess potential hematological and vascular causes of thromboembolism. The lower limb Doppler report was negative, and the thrombophilia screen also had negative results, effectively ruling out hematological and vascular causes for this massive PE.

On the third day, he was transferred to the high dependency unit (HDU), where heparin was discontinued, and Clexane 90 mg twice daily was initiated. Oxygen therapy was reduced to 1 L of NC. He remained stable without active complaints.

On the fourth day, he was transferred to the medical ward for two days for close monitoring by the medical department, where the patient showed great improvement and started to maintain oxygen on room air. Moreover, he was seen by the physiotherapy team for rehabilitation after PE. Clexane was later switched to apixaban to change its therapeutic low molecular weight heparin (enoxaparin 90 mg subcutaneously twice daily).

On the fifth day, the patient was discharged in stable condition with no active complaints. His discharge vitals were blood pressure of 136/72 mmHg, heart rate of 81 bpm, temperature of 36.9°C, respiratory rate of 20, and oxygen saturation of 98% on room air. He was advised to continue apixaban 5 mg twice daily.

During follow-up visits at two weeks, one month, three months, six months, and one year post-discharge, the patient consistently reported minimal exertional symptoms that are getting better with time, and he is back to his normal lifestyle. Comprehensive testing, including a CBC, blood film, coagulation profile, renal function test (RFT) and liver function test (LFT), viral serology, ANA profile, antiphospholipid (APS) antibodies, tumor markers, and genetic screening for factor V Leiden (FVL) and prothrombin mutations, showed a negative result. This effectively ruled out any underlying causes of PE. Additionally, a partial thrombophilia screen was negative.

The patient's follow-up care involved thorough evaluations by both a hematologist and a pulmonologist, which confirmed no recurrence. By the time of his most recent internal medicine clinic visit, the patient demonstrated significant improvement and remained symptom-free, marking a successful recovery from the initial life-threatening event.

## Discussion

It is important to consider PE in the differential diagnosis when encountering relevant clinical symptoms due to its potentially life-threatening consequences [11]. Around one-third of people with DVT or PE experience a recurrence within a decade. Blood clots impact nearly 900,000 Americans each year and cause roughly 100,000 deaths, with half of these tied to healthcare settings [12].

Recent studies have demonstrated that new biomarkers, such as galectin-3, have significant diagnostic value and are accurate in identifying acute PE in the emergency department [10]. A study by the American College of Physicians highlights the difficulties in diagnosing PE and cautions against overusing tests such as CT scans and D-dimer, which can pose risks and lead to high costs. Instead, it recommends using clinical tools to diagnose PE before ordering tests. For patients with a low probability, avoiding unnecessary testing is key. In cases where the probability is moderate, high-sensitivity D-dimer tests are recommended, with adjusted thresholds for older patients; for patients with a high probability of PE, CT angiography or ventilation-perfusion scans remain the best options for diagnosis [13]. Diagnosing PE presents significant challenges, including non-specific symptoms, variable clinical presentations, and limitations in diagnostic tests. At the same time, a recent study also highlighted that emergency physicians and facilitators in the outpatient management of low-risk PE in the United States face key barriers. Emergency departments emphasize the role of clinician beliefs, fears, and local culture, along with external factors such as medicolegal climate and insurance status [14]. Patients suffering from PE need treatment with anticoagulants. Traditionally, those with cancer and blood clots were treated with low-molecular-weight heparin (LMWH). However, recent studies involving three-factor Xa inhibitors have demonstrated that non-vitamin K oral anticoagulants (NOACs) are as effective as LMWH, as shown in four randomized clinical trials [15]. Correcting blood pressure in PE patients is crucial to preventing hemodynamic collapse, ensuring vital organ perfusion, reducing the risk of cardiac arrest, and enhancing the effectiveness of PE treatments [16]. In the emergency department, critical thinking and quick spot diagnosis are crucial, and it is always important to have a differential diagnosis based on the symptoms and associated lab results. The Pulmonary Embolism Comprehensive Screening Score (PECSS) is designed for emergency departments to safely rule out PE in suspected cases, streamlining the diagnostic process and enhancing patient safety [17].

Our patient was presented to the emergency department after collapsing at home with symptoms of dizziness, shortness of breath, and sweating. Upon examination, he was found to have bradycardia and hypotension, which improved following the ABCDE approach, intravenous fluid administration, and 2 L of oxygen via NC. The diagnosis of PE was confirmed by CT pulmonary angiogram. He was admitted to the hospital for five days and placed on a strict management protocol, including thrombolytic therapy with tissue plasminogen activator (tPA), after confirming no contraindications. Post-TPA, he showed no signs of active bleeding or recurrence of symptoms.

He subsequently underwent a 48-hour heparin infusion in the ICU where he remained for the first 24 hours before being transferred to the HDU for an additional 24 hours. Throughout his stay, vigilant monitoring of APTT and any potential bleeding complications was conducted. His anticoagulation therapy was then switched to Clexane 90 mg twice daily. Upon discharge, his medication was changed to apixaban 10 mg twice daily, which was eventually tapered to 5 mg twice daily. He returned for multiple visits to the internal medicine clinic after his initial admission, where tests were assured to rule out any genetic disorders or tumors.

## Conclusions

This case report highlights a 37-year-old male with massive bilateral PE, remarkable for the absence of typical risk factors. It underscores the critical importance of prompt diagnosis and tailored treatment in managing life-threatening conditions such as PE. Timely imaging and intervention play an important role, particularly for high-risk patients, enabling rapid initiation of life-saving therapies such as thrombolysis or surgery when necessary. For more stable cases, thorough diagnostic testing and individualized treatment plans, including oral anticoagulants or blood thinners, are key to effective management. This case also emphasizes the role of organized timed follow-up to monitor post-PE symptoms and improvement, and it is also important to avoid recurrence, with a reminder to the patient regarding the importance of following the medications as prescribed.
